# An *in Vivo* Mouse Model to Investigate the Effect of Local Anesthetic Nanomedicines on Axonal Conduction and Excitability

**DOI:** 10.3389/fnins.2018.00494

**Published:** 2018-07-26

**Authors:** Mihai Moldovan, Susana Alvarez, Christian Rothe, Thomas L. Andresen, Andrew Urquhart, Kai H. W. Lange, Christian Krarup

**Affiliations:** ^1^Department of Neuroscience, University of Copenhagen, Copenhagen, Denmark; ^2^Department of Clinical Neurophysiology, Rigshospitalet, Copenhagen, Denmark; ^3^Department of Anesthesia, Nordsjællands Hospital, Hillerød, Denmark; ^4^Department for Micro- and Nanotechnology, Technical University of Denmark, Lyngby, Denmark

**Keywords:** liposomes, peripheral nerve block, threshold-tracking, *in vivo* imaging, lidocaine

## Abstract

Peripheral nerve blocks (PNBs) using local anesthetic (LA) are superior to systemic analgesia for management of post-operative pain. An insufficiently short PNB duration following single-shot LA can be optimized by development of extended release formulations among which liposomes have been shown to be the least toxic. *In vivo* rodent models for PNB have focused primarily on assessing behavioral responses following LA. In a previous study in human volunteers, we found that it is feasible to monitor the effect of LA *in vivo* by combining conventional conduction studies with nerve excitability studies. Here, we aimed to develop a mouse model where the same neurophysiological techniques can be used to investigate liposomal formulations of LA *in vivo*. To challenge the validity of the model, we tested the motor PNB following an unilamellar liposomal formulation, filled with the intermediate-duration LA lidocaine. Experiments were carried out in adult transgenic mice with fluorescent axons and with fluorescent tagged liposomes to allow *in vivo* imaging by probe-based confocal laser endomicroscopy. Recovery of conduction following LA injection at the ankle was monitored by stimulation of the tibial nerve fibers at the sciatic notch and recording of the plantar compound motor action potential (CMAP). We detected a delayed recovery in CMAP amplitude following liposomal lidocaine, without detrimental systemic effects. Furthermore, CMAP threshold-tracking studies of the distal tibial nerve showed that the increased rheobase was associated with a sequence of excitability changes similar to those found following non-encapsulated lidocaine PNB in humans, further supporting the translational value of the model.

## Introduction

Adequate pain management has been shown to improve the rate and quality of patient recovery following surgery (Zaslansky et al., 2015). Peripheral nerve blocks (PNBs) using local anesthetic (LA) are superior to opioid analgesia which have a high rate of undesirable systemic effects (Wu and Raja, 2011; Aguirre et al., 2012). Voltage-gated Na+ channel (VGSC) blockers acting as LA (e.g. bupivacaine, lidocaine, etc.) are the most widely used drugs in PNBs. Single PNB only lasts several hours (Wu and Raja, 2011). Catheter-based PNBs using LA infusions can extend analgesia (Madsen et al., 2018); however, their efficiency is limited by mechanical factors and can expose patients to large quantities of LA (Ilfeld, 2017). Alternatively, several LA carriers with extended release have been developed, among which liposomes have been shown to be the least toxic (Weiniger et al., 2012). A multilamellar liposome formulation of bupivacaine (Exparel) has so far failed to show PNB of major nerves (Hadzic et al., 2016) and remains of limited indication (Vyas et al., 2016). Further experimental work is required to understand the *in vivo* effects of liposomal LA nanomedicines.

*In vivo* rodent models for PNB have focused primarily on assessing behavioral responses following LA such as the motor performance on an inverted mesh (Leszczynska and Kau, 1992), the tail flick latency to a thermal stimulus (Grant et al., 1993), or the vocalization responses to an electrical shock (Grant et al., 2000). Such sensory-motor behavioral techniques were successfully used to investigate the protracted effect of liposomal LA (Epstein-Barash et al., 2009), and nevertheless, the effect on axonal function remains poorly investigated. In a previous study in human volunteers, we found that it is feasible to monitor the effect of LA *in vivo* by combining conventional conduction studies with nerve excitability studies by threshold-tracking (Moldovan et al., 2014). Here, we aimed to develop a translational mouse model where the same neurophysiological techniques can be used to investigate liposomal formulations of LA *in vivo*. To challenge the validity of the model, we tested an unilamellar liposomal formulation, consisting of a single lipid bilayer (Betageri and Parsons, 1992) which has faster release profile than multilamellar liposomes (Silva et al., 2016). The liposomes were filled with the LA lidocaine which has a shorter duration of action than bupivacaine in extended-release formulations (Huynh et al., 2015).

## Materials and Methods

### Mice, Anesthesia, and Experimental Design

Investigations *in vivo* were carried out in adult mice of C57Bl background (3- to 5-month-old) with axonal expression of the Yellow Fluorescent Protein (YFP) reporter (homozygote Thy1-YFP) obtained from the Jackson Laboratory, Bar Harbor, ME, United States. General anesthesia was ensured using a 1:1 mixture of Hypnorm/Midazolam (5 mg/ml). A volume of 0.1 ml/10 g from the mixture was injected subcutaneously for induction, and then maintained with 50% hourly for up to 4 h as needed.

**FIGURE 1:**
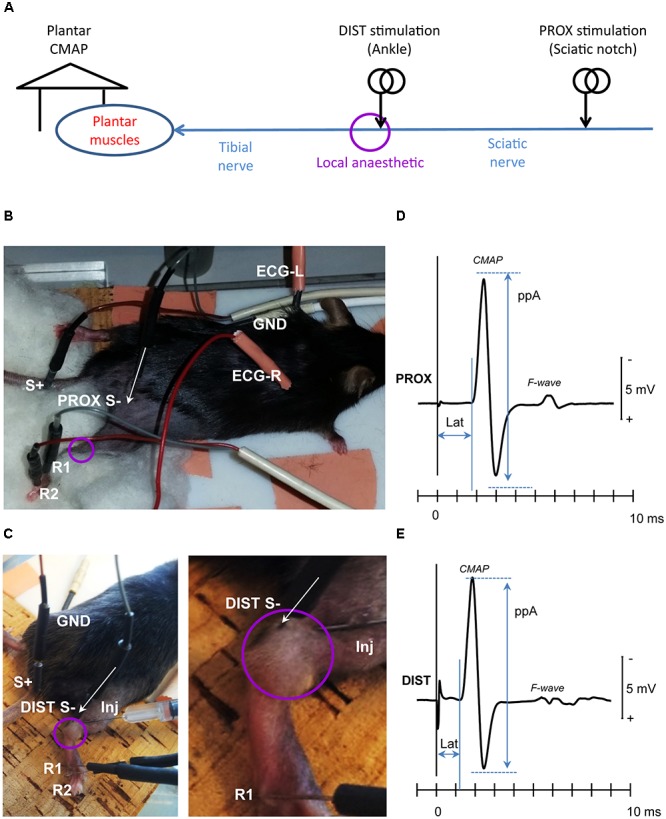
Experimental setup. **(A)** Tibial nerve fibers were stimulated proximally (PROX) at the sciatic notch of the ilium (sciatic nerve), to assess recovery of conduction across the local anesthesia (LA) site, and distally (DIST) to assess recovery of tibial nerve excitability. The evoked compound muscle action potential (CMAP) was recorded from the plantar muscles. **(B)** Needle electrode placement for PROX stimulation (S-, S+), CMAP recording (R1, R2), ground electrode (GND), and ECG recording (ECG-R, ECG-L); **(C)** Needle electrode placement for DIST stimulation (S-, S+), CMAP recording (R1, R2), and the ground electrode (GND). The detail at right depicts the local anesthetic injection needle (Inj) as well as the bubble engulfing the tibial nerve at the ankle. The baseline CMAP recordings evoked from the PROX and DIST stimulation sites were depicted in panels **(D,E)**, respectively. The amplitude CMAP was measured peak-to-peak (ppA) and the corresponding latency (Lat) to the first take-off from baseline. Note that from both stimulation sites, the evoked CMAP was followed by a secondary motor response (F-wave) resulting from antidromic activation of the motor neuron pool.

**FIGURE 2:**
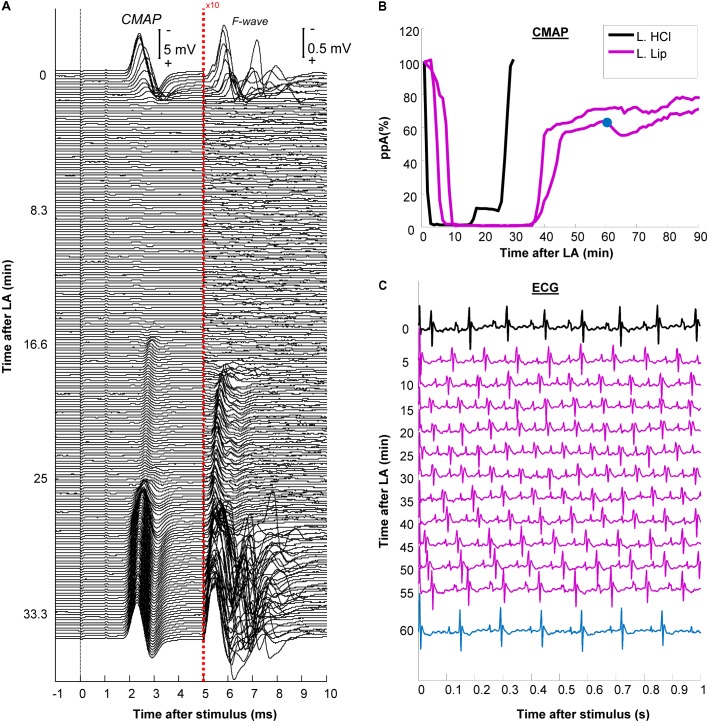
Development and recovery of conduction block after injection of the local anesthetic (LA). **(A)** The plantar compound muscle action potential CMAP evoked by proximal stimulation at the sciatic notch after non-encapsulated lidocaine (L. HCL) exposure. After 5 ms, the recordings are presented at 10-fold larger amplitude to facilitate the visualization of F-waves; **(B)** The relative changes in peak-to-peak CMAP amplitude (ppA) were shown for the non-encapsulated lidocaine (L. HCl) experiment in **(A)** versus 2 other different experiments using liposomal lidocaine (L. Lip); **(C)** ECG traces recorded during exposure to liposomal lidocaine. Note that the heart rate, measured from the QRS complexes, remained between 8 and 10 s (corresponding to 480–600 beats per minute). The ppA recovery corresponding to the ECG after 60 min is indicated in blue in panel **(B)**.

**FIGURE 3:**
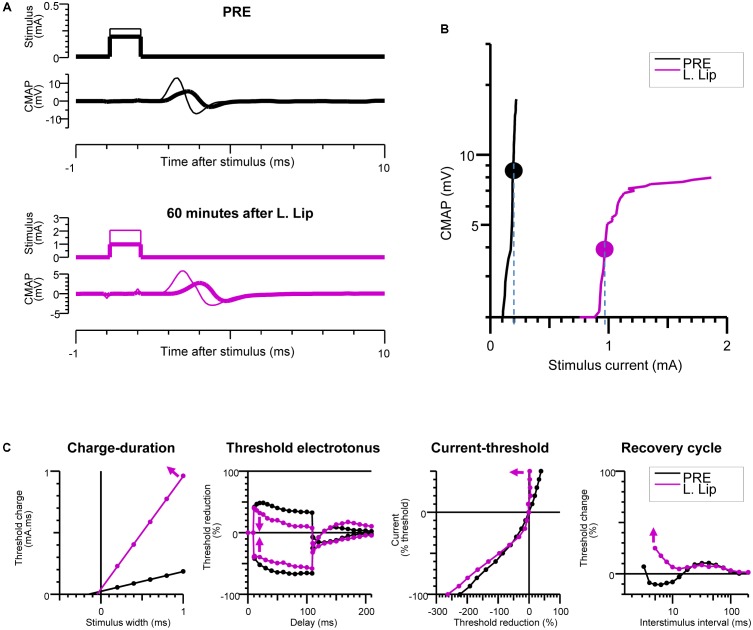
Recovery of excitability after injection of the local anesthetic (LA). Baseline measurements (PRE) are compared with measurements at 60 min after liposomal lidocaine (L. Lip) when there was a partial recovery of conduction. **(A)** Plantar compound motor action potentials (CMAPs) were evoked by distal stimulation of the tibial nerve at the ankle (within the nerve area exposed to LA). The thick traces indicate the CMAPs closest to 40% of the maximal CMAP amplitude, referred to as threshold CMAPs; **(B)** The stimulus-response measurements corresponding to the recordings in **(A)**. The stippled lines indicate the current required to evoke the threshold CMAP (symbols), referred to as threshold; **(C)** The multiple measures of nerve excitability by “threshold-tracking” protocol, measuring the changes in threshold when varying stimulus duration (charge–duration), when conditioning with 100 ms depolarizing (upward) and hyperpolarizing (downward) currents that were set to 40% of threshold (threshold electrotonus), when conditioning with a range of 200 ms polarizing currents that were stepped from +50% (top) to –100% (bottom) of threshold (current–threshold relationship) and following a supramaximal conditioning stimulus (recovery cycle). The arrows indicate that following liposomal lidocaine there was: an increase in the charge–duration slope, a reduction in threshold changes during both depolarizing and hyperpolarizing threshold electrotonus, a marked increase in the current–threshold slope on depolarization, and an increased in refractoriness of the recovery cycle.

Using *in vivo* neurophysiological recordings (**Figure 1**), we monitored the recovery of tibial nerve conduction (**Figure 2**) and excitability (**Figure 3**) following injection of the LA. Slow subcutaneous injection of a volume of 50 μl at the ankle engulfed the tibial nerve in an easily observable bubble (**Figure 1C**) ensuring the reproducibility of the LA exposure without the need of ultrasound visualization as in the case of human PNBs (Moldovan et al., 2014). The monitoring of the systemic toxicity was carried out using a 1-channel electrocardiogram (ECG) recorded between the forepaws (**Figure 1B**) (MP150 with an ECG100C module, Biopac Systems Inc., United States).

**FIGURE 4:**
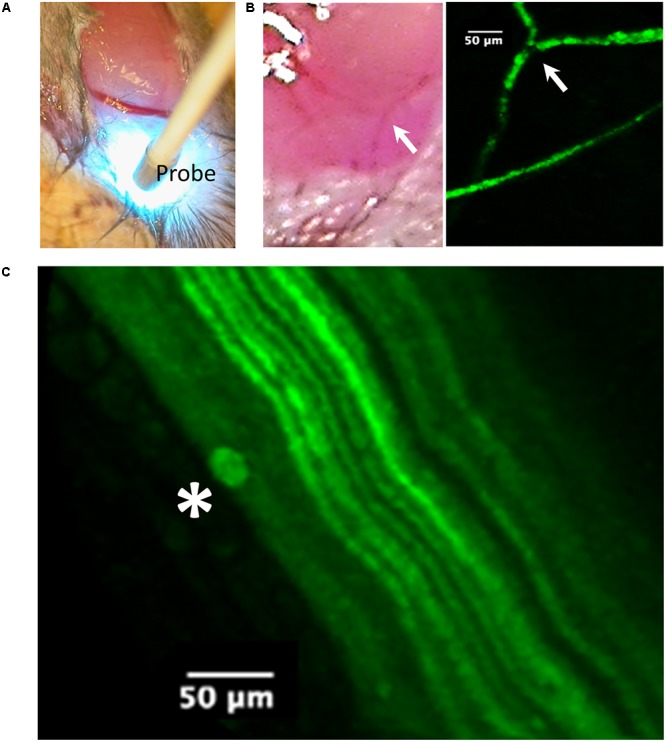
Imaging of the tibial nerve block area by probe-based confocal laser endomicroscopy (pCLE) at 90 min after liposomal lidocaine (L. Lip). **(A)** The ankle was first dissected to allow access of the imaging probe (Probe) to the subcutaneous tissue. Note the scattering of the blue laser light used for imaging; **(B)** Superficial veins were fluorescent consistent with the absorption of the fluorescent liposomes. The arrow identifies the same venous confluence in light imaging and pCLE imaging; **(C)** With further advancement of the imaging probe between the muscle planes (blunt dissection), the tibial nerve could be readily identified due to its fluorescent fibers (axonal YFP reporter expression). Occasionally, liposomal accumulations (star) could be identified near the fibers.

At the completion of the electrophysiological experiments, the lateral aspect of the leg was exposed for imaging studies (**Figure 4**) after which the mice were killed by cervical dislocation.

This study was carried out in accordance with the recommendations of directive 2010/63/EU of the European parliament and of the council on the protection of animals used for scientific purposes. The protocol was approved by the Danish Animal Experiments Inspectorate.

### Electrophysiological Setup

For *in vivo* electrophysiological investigations, the mice were placed on a temperature-controlled pad (HB 101/2, LSI Letica) set to 37°C (Moldovan and Krarup, 2006). Stimulation and recording were carried out using percutaneously inserted Pt-needle electrodes (Moldovan and Krarup, 2006; Wild et al., 2018). Electrical stimuli generated from a constant current stimulator (DS4, Digitimer Ltd.) were delivered proximally to the sciatic nerve at the sciatic notch (**Figures 1A,B**) and distal to the tibial nerve at the ankle (**Figures 1A,C**). The evoked compound muscle potential (CMAP) of the plantar muscle was recorded using a bandpass filter of 10 Hz–6 kHz (Neurolog NL820A amplifier with NL844 Pre-Amplifier, Digitimer Ltd., United Kingdom). A ground reference electrode was inserted into the back of the mouse. The near-nerve electrode placement was carried out ensuring the lowest threshold current.

The signals were digitized into a PC-based recording and control system via a multifunction I/O device (PCI-6221, National Instruments Corporation Ltd., United Kingdom). The amplitudes of the CMAP evoked from the proximal (**Figure 1D**) and distal (**Figure 1E**) stimulation sites were measured peak-to-peak (ppA). The CMAP latency was measured to the first take-off from baseline (**Figures 1D,E**).

It should be noted that both from the proximal stimulation site (**Figure 1D**) as well as from the distal stimulation site (**Figure 1E**), the evoked CMAP was followed by a secondary motor response (“F-wave”) resulting from motoneuronal backfiring following antidromic activation in humans (Magladery and Mcdougal, 1950) and rodents (Meinck, 1976; Robertson et al., 1993; Moldovan et al., 2012). Although the F-waves were not specifically quantified, their presence was considered as an indication of the functional integrity of the motoneurons and the alpha-motor fibers.

### Nerve Conduction Studies

We have previously established a setup for the continuous monitoring of tibial nerve conduction by stimulation at the ankle for up to 4 h (Moldovan and Krarup, 2006; Alvarez et al., 2008). Nevertheless, overcoming the partial voltage-gate Na+ channel block by lidocaine could require very large stimulation currents (Moldovan et al., 2014). Given the small distances, using such high currents at ankle could cause the stimulus to” jump” and activate the unanesthetized part of the tibial nerve. To avoid this potential confounder, we tested the changes in conduction across the PNB by proximal stimulation (**Figures 1A,B**). Stimulation and recording were controlled using a custom control software developed in MATLAB (version R2013b, MathWorks Inc., United States). We delivered negative rectangular stimuli of 1 ms duration at 0.5 Hz and recorded 1000 ms sweeps with a sampling frequency of 125 KHz allowing synchronized CMAP (**Figure 2A**) and ECG recordings (**Figure 2C**). The amplitudes at proximal stimulation were averaged over 1-min and expressed, relative to the 1st minute prior to injection (**Figure 2B**). The stimulation current was set at twofold the current required to obtain a 100% ppA CMAP and kept constant for the entire duration of the recording.

### Nerve Excitability Studies

The changes in nerve excitability at the PNB site were monitored by distal stimulation at the ankle (**Figures 1A,C**) and tracking changes in the threshold current (**Figures 3A,B**) required to evoke a 40% ppA CMAP (Bostock et al., 1998) using QtracS stimulation software for recording and QtracP software for analysis (©Institute of Neurology, London, United Kingdom). We used that same TRONDH multiple excitability protocol (Kiernan and Bostock, 2000), that we used in our human PNB study (Moldovan et al., 2014). We have previously adapted this protocol for use in mice (Moldovan et al., 2009, 2011). This allowed recording of the full sequence of excitability measures (**Figure 3C**) as previously described in detail (Bostock et al., 1998; Kiernan and Bostock, 2000; Kiernan et al., 2000): charge–duration relationship to measure the rheobase, threshold electrotonus to measure accommodation to shifts in depolarizing and hyperpolarizing membrane potential, current–threshold relationship to measure input conductance, and the recovery of excitability following conduction of the action potential to measure refractoriness.

### Imaging Studies

*In vivo* imaging (**Figure 4**) was carried out using probe-based confocal laser endomicroscopy (pCLE) using a Cellvizio single band, with a 488-nm laser (Mauna Kea Technologies, France) which allows for the investigation of fluorescent blood vessels and axons (Wong et al., 2009). The frames were captured via a S-1500 objective (**Figure 4A**) and exported using the Cellvizio Viewer for PC (version 1.4.2).

### Liposomal LA Formulation

We formed an unilamelar liposomes using the established methods of freeze drying lipids followed by rehydration in lidocaine hydrochloride (2% w/v) phosphate-buffered saline. This solution was pressure extruded through 100 nm filters to reduce liposome size polydispersity. The liposomes comprised of hydrogenated soy L-α-phosphatidylcholine (HSPC), cholesterol (Chol), 1,2-distearoyl-sn-glycero-3-phosphoethanolamine-N-methoxy (polyethylene glycol)-2000 (DSPE-PEG2000), and 1-palmitoyl-2-(dipyrrometheneboron difluoride)undecanoyl-sn-glycero-3-phosphocholine (TopFluor PC) in a mol% ratio of 54.8:40:5:0.2, respectively. Polyethylene glycol conjugated lipid was introduced into the liposome bilayer to increase residency time (Eriksen et al., 2017) and TopFluor PC was incorporated to allow visualization. The resulting liposome size (Mean ± SD) was found to be 196.2 ± 2.2 nm with a size polydispersity index of 0.134 ± 0.03 as determined by dynamic light scattering. A non-encapsulated lidocaine salt solution (20 mg/ml, L. HCl) served as control. All chemical components were purchased from commercial suppliers (Sigma–Aldrich, DE, and Avanti Polar Lipids, United States).

## Results

### Recovery of Conduction

Proximal stimulation of the tibial nerve fibers at the sciatic notch (**Figures 1A,B**) evoked a CMAP (Mean ± SD) with a ppA of 12 ± 4 mV and a latency of 1.7 ± 0.1 ms (**Figure 1D**). Distal stimulation of the tibial nerve fibers at the ankle (**Figures 1A,C**) evoked a CMAP (Mean ± SD) with a ppA of 16 ± 5 mV and a latency of 1.2 ± 0.1 ms (**Figure 1E**). Note that the proximal CMAP was not larger than the distal CMAP indicating that the CMAP measurements reflected tibial-nerve innervated muscles with minimal contamination (if any) from common peroneal nerve innervated muscles.

An experiment with non-encapsulated lidocaine is detailed in **Figure 2A**. Within 3 min following LA injection, there was a complete abolishment of the CMAP and F-waves evoked by proximal stimulation which recovered completely within 30 min (**Figure 2A**). The time-course of CMAP amplitude (ppA) recovery, measured relative to the minute before LA, is presented in **Figure 2B**. In contrast, our model was able to detect a slower recovery after liposomal lidocaine, with good reproducibility in repeated studies (**Figure 2B**). Furthermore, we found that following liposomal lidocaine the CMAP evoked by proximal stimulation recovered rapidly to above 50% ppA whereas the subsequent recovery was much slower (**Figure 1B**), so that a full recovery was observed after 3 h (data not shown). Although liposomes were absorbed, as indicated by pCLE (**Figure 4B**), there were no ECG signs of bradycardia (**Figure 2C**) that could indicate a confounding effect of lidocaine toxicity.

### Recovery of Excitability

During the slow CMAP recovery phase after liposomal lidocaine (**Figure 2B**), liposomal accumulations could be demonstrated by pCLE near the tibial nerve fibers at the ankle (**Figure 4C**) suggesting a persistent release. An experiment with uncaged lidocaine is detailed in **Figure 3**. We found that during the partial CMAP recovery phase, evoking the maximal CMAP by distal stimulation in the nerve exposed to LA required a much larger stimulation current (**Figure 3A**) with a right-shift in the stimulus-response curve (**Figure 3B**). At that time, the current required to evoke the CMAP from the proximal stimulation site remained undistinguishable from the value prior to LA (data not shown).

To explore the mechanisms of impaired tibial nerve excitability at the LA site, we carried out multiple measures of excitability by “threshold-tracking” (**Figure 3C**). Consistent with the larger stimulus current required to evoke the CMAP, we found a marked increase in rheobase (Weiss, 1901; Bostock et al., 1998), as indicated by the slope of the charge–duration relationship (**Figure 3C**). Moreover, the observed increase in rheobase after liposomal lidocaine was associated with (**Figure 3C**) a reduction in threshold changes during both depolarizing and hyperpolarizing threshold electrotonus, a marked increase in the current–threshold slope (input conductance) on depolarization, and an increased in refractoriness of the recovery cycle (**Figure 3C**).

## Discussion

We developed an *in vivo* mouse model to explore the effect of local anesthetic nanomedicines on axonal function. We tested the validity of the model by comparing the effect of a liposomal lidocaine formulation versus non-encapsulated lidocaine on motor axon function and found a slower conduction recovery along the tibial nerve following liposomal lidocaine. Furthermore, the model opened the possibility to combine conduction studies with “threshold-tracking” excitability studies in the same nerve, allowing a translational mechanistic insight into the liposomal release *in vivo*.

Assessment of the effect of liposomal local anesthetic nanomedicines on sciatic nerve block in rodents has been previously carried out previously by behavioral measures (Yin et al., 2016). The sensory (thermal nociceptive) blockade was investigated in a hot-plate paradigm (Sagie and Kohane, 2010), whereas the motor blockade was investigated using the extensor postural thrust test (Sagie and Kohane, 2010). Although these behavioral studies provided a valuable approach to distinguish the motor versus sensory selectivity of the nanomedicine, they offered little information on the actual changes in axonal function. It is long known that the susceptibility to an anesthetic conduction block is different between neuronal populations, reflecting differences in membrane properties (Gasser and Erlanger, 1929). Nevertheless, motor population was found to be at least as good an indicator for changes in the duration of PNB as the sensory fibers (Dietz and Jaffe, 1997; Gokin et al., 2001) even though they do not directly reflect the analgesic effect. Furthermore, the fact that the myelinated motor axon population is more functionally homogenous than the sensory fiber population (Gasser and Erlanger, 1929), can convey an advantage in characterizing the excitability changes.

The LA lidocaine is thought to impair the function of the voltage-gated Na^+^ channels (VGSC) (Sheets and Hanck, 2007) reducing the axonal “safety factor” for conduction (Tasaki, 1953) in excitable tissues of the nervous system (Nathan and Sears, 1961) as well as the heart (Austen and Moran, 1965). Although the pCLE imaging studies indicated liposome accumulation within veins (**Figure 4B**), the ECG (**Figure 2C**) remained within normal limits (Ho et al., 2011). We therefore do not think that the observed effects on axonal function were confounded by lidocaine cardiotoxicity (Fox and Kenmore, 1967).

We confirmed an increase in the threshold for electrical stimulation during recovery after LA (Gasser and Erlanger, 1929; Gasser and Grundfest, 1939). Similar deviations in excitability were found in our previous study using non-encapsulated lidocaine in humans (Moldovan et al., 2014) which supports the translational value of our mouse model. These excitability deviations differed from those observed with selective VGSC block following accidental tetrodotoxin poisoning in humans (Kiernan et al., 2005). It is likely that the protracted period of reduced nerve excitability following LA (Tabatabai and Booth, 1990) reflected both the partial recovery of the Na^+^ conductance as well as an impairment of the axolemmal passive electrical properties (Moldovan et al., 2014) due to a physical effect of lidocaine dissolved within the membrane (Kassahun et al., 2010). In further studies, excitability changes could allow the detection of early signs of toxicity (Moldovan et al., 2009) in combination with imaging of morphological changes (Beirowski et al., 2005; Alvarez et al., 2008, 2013).

## Author Contributions

CK and KL initiated the study. MM made the neurophysiological control software. AU made the liposomal lidocaine formulation. All the authors contributed to the experiments, analyzed the data, discussed the results, and wrote the manuscript.

## Conflict of Interest Statement

The authors declare that the research was conducted in the absence of any commercial or financial relationships that could be construed as a potential conflict of interest.

## References

[B1] Aguirre J. Del Moral A. Cobo I. Borgeat A. Blumenthal S. (2012). The role of continuous peripheral nerve blocks. *Anesthesiol. Res. Pract.* 2012:560879. 10.1155/2012/560879 22761615 PMC3385590

[B2] Alvarez S. Moldovan M. Krarup C. (2008). Acute energy restriction triggers Wallerian degeneration in mouse. *Exp. Neurol.* 212 166–178. 10.1016/j.expneurol.2008.03.022 18486130

[B3] Alvarez S. Moldovan M. Krarup C. (2013). Prolonged high frequency electrical stimulation is lethal to motor axons of mice heterozygously deficient for the myelin protein P(0) gene. *Exp. Neurol.* 247 552–561. 10.1016/j.expneurol.2013.02.006 23439028

[B4] Austen W. G. Moran J. M. (1965). Cardiac and peripheral vascular effects of lidocaine and procainamide. *Am. J. Cardiol.* 16 701–707. 10.1016/0002-9149(65)90054-8 5837148

[B5] Beirowski B. Adalbert R. Wagner D. Grumme D. S. Addicks K. Ribchester R. R. (2005). The progressive nature of Wallerian degeneration in wild-type and slow Wallerian degeneration (WldS) nerves. *BMC Neurosci.* 6:6. 10.1186/1471-2202-6-6 15686598 PMC549193

[B6] Betageri G. V. Parsons D. L. (1992). Drug encapsulation and release from multilamellar and unilamellar liposomes. *Int. J. Pharm.* 81 235–241. 10.1016/0378-5173(92)90015-T

[B7] Bostock H. Cikurel K. Burke D. (1998). Threshold tracking techniques in the study of human peripheral nerve. *Muscle Nerve* 21 137–158. 10.1002/(SICI)1097-4598(199802)21:2<137::AID-MUS1>3.0.CO;2-C 9466589

[B8] Dietz F. B. Jaffe R. A. (1997). Bupivacaine preferentially blocks ventral root axons in rats. *Anesthesiology* 86 172–180. 10.1097/00000542-199701000-00021 9009952

[B9] Epstein-Barash H. Shichor I. Kwon A. H. Hall S. Lawlor M. W. Langer R. (2009). Prolonged duration local anesthesia with minimal toxicity. *Proc. Natl. Acad. Sci. U.S.A.* 106 7125–7130. 10.1073/pnas.0900598106 19365067 PMC2678453

[B10] Eriksen A. Z. Brewer J. Andresen T. L. Urquhart A. J. (2017). The diffusion dynamics of PEGylated liposomes in the intact vitreous of the ex vivo porcine eye: a fluorescence correlation spectroscopy and biodistribution study. *Int. J. Pharm.* 522 90–97. 10.1016/j.ijpharm.2017.03.003 28267579

[B11] Fox J. L. Kenmore P. I. (1967). The effect of ischemia on nerve conduction. *Exp. Neurol.* 17 403–419. 10.1016/0014-4886(67)90127-6 6020656

[B12] Gasser H. S. Erlanger J. (1929). The role of fiber size in the establishment of a nerve block by pressure or cocaine. *Am. J. Physiol.* 88 581–591. 10.1152/ajplegacy.1929.88.4.581

[B13] Gasser H. S. Grundfest H. (1939). Axon diameters in relation to the spike dimensions and the conduction velocity in mammalian A fibers. *Am. J. Physiol.* 127 393–414. 10.1152/ajplegacy.1939.127.2.393

[B14] Gokin A. P. Philip B. Strichartz G. R. (2001). Preferential block of small myelinated sensory and motor fibers by lidocaine: in vivo electrophysiology in the rat sciatic nerve. *Anesthesiology* 95 1441–1454. 10.1097/00000542-200112000-00025 11748404

[B15] Grant G. J. Piskoun B. Lin A. Bansinath M. (2000). An in vivo method for the quantitative evaluation of local anesthetics. *J. Pharmacol. Toxicol. Methods* 43 69–72. 10.1016/S1056-8719(00)00079-4 11091131

[B16] Grant G. J. Zakowski M. I. Vermeulen K. Langerman L. Ramanathan S. Turndorf H. (1993). Assessing local anesthetic effect using the mouse tail flick test. *J. Pharmacol. Toxicol. Methods* 29 223–226. 10.1016/1056-8719(93)90029-E 8400418

[B17] Hadzic A. Minkowitz H. S. Melson T. I. Berkowitz R. Uskova A. Ringold F. (2016). Liposome bupivacaine femoral nerve block for postsurgical analgesia after total knee arthroplasty. *Anesthesiology* 124 1372–1383. 10.1097/ALN.0000000000001117 27035853

[B18] Ho D. Zhao X. Gao S. Hong C. Vatner D. E. Vatner S. F. (2011). Heart rate and electrocardiography monitoring in mice. *Curr. Protoc. Mouse Biol.* 1 123–139. 10.1002/9780470942390.mo100159 21743842 PMC3130311

[B19] Huynh T. M. Marret E. Bonnet F. (2015). Combination of dexamethasone and local anaesthetic solution in peripheral nerve blocks: a meta-analysis of randomised controlled trials. *Eur. J. Anaesthesiol.* 32 751–758. 10.1097/EJA.0000000000000248 25774458

[B20] Ilfeld B. M. (2017). Continuous peripheral nerve blocks: an update of the published evidence and comparison with novel, alternative analgesic modalities. *Anesth. Analg.* 124 308–335. 10.1213/ANE.0000000000001581 27749354

[B21] Kassahun B. T. Murashov A. K. Bier M. (2010). A thermodynamic mechanism behind an action potential and behind anesthesia. *Biophys. Rev. Lett.* 5 35–41. 10.1142/S1793048010001123

[B22] Kiernan M. C. Bostock H. (2000). Effects of membrane polarization and ischaemia on the excitability properties of human motor axons. *Brain* 123 2542–2551. 10.1093/brain/123.12.2542 11099455

[B23] Kiernan M. C. Burke D. Andersen K. V. Bostock H. (2000). Multiple measures of axonal excitability: a new approach in clinical testing. *Muscle Nerve* 23 399–409. 10.1002/(SICI)1097-4598(200003)23:3<399::AID-MUS12>3.0.CO;2-G 10679717

[B24] Kiernan M. C. Isbister G. K. Lin C. S. Burke D. Bostock H. (2005). Acute tetrodotoxin-induced neurotoxicity after ingestion of puffer fish. *Ann. Neurol.* 57 339–348. 10.1002/ana.20395 15732107

[B25] Leszczynska K. Kau S. T. (1992). A sciatic nerve blockade method to differentiate drug-induced local anesthesia from neuromuscular blockade in mice. *J. Pharmacol. Toxicol. Methods* 27 85–93. 10.1016/1056-8719(92)90026-W 1591408

[B26] Madsen M. H. Christiansen C. B. Rothe C. Andreasen A. M. Lundstrom L. H. Lange K. H. W. (2018). Local anesthetic injection speed and common peroneal nerve block duration: a randomized controlled trial in healthy volunteers. *Reg. Anesth. Pain Med.* 43 467–473. 10.1097/AAP.0000000000000759 29570501

[B27] Magladery J. W. Mcdougal D. B. (1950). Electrophysiological studies of nerve and reflex activity in normal man. I. Identification of certain reflexes in the electromyogram and the conduction velocity of peripheral nerve fibres. *Bull. Johns Hopkins Hosp.* 86 265–290. 15414383

[B28] Meinck H. M. (1976). Occurrence of the H reflex and the F wave in the rat. *Electroencephalogr. Clin. Neurophysiol.* 41 530–533. 10.1016/0013-4694(76)90064-X 61856

[B29] Moldovan M. Alvarez S. Krarup C. (2009). Motor axon excitability during Wallerian degeneration. *Brain* 132(Pt 2), 511–523. 10.1093/brain/awn332 19074190

[B30] Moldovan M. Alvarez S. Pinchenko V. Klein D. Nielsen F. C. Wood J. N. (2011). Nav1.8 channelopathy in mutant mice deficient for myelin protein zero is detrimental to motor axons. *Brain* 134(Pt 2), 585–601. 10.1093/brain/awq336 21169333

[B31] Moldovan M. Alvarez S. Pinchenko V. Marklund S. Graffmo K. S. Krarup C. (2012). Nerve excitability changes related to axonal degeneration in amyotrophic lateral sclerosis: insights from the transgenic SOD1(G127X) mouse model. *Exp. Neurol.* 233 408–420. 10.1016/j.expneurol.2011.11.008 22116045

[B32] Moldovan M. Krarup C. (2006). Evaluation of Na+/K+ pump function following repetitive activity in mouse peripheral nerve. *J. Neurosci. Methods* 155 161–171. 10.1016/j.jneumeth.2005.12.015 16466807

[B33] Moldovan M. Lange K. H. Aachmann-Andersen N. J. Kjaer T. W. Olsen N. V. Krarup C. (2014). Transient impairment of the axolemma following regional anaesthesia by lidocaine in humans. *J. Physiol.* 592(Pt 13), 2735–2750. 10.1113/jphysiol.2014.270827 24710060 PMC4221817

[B34] Nathan P. W. Sears T. A. (1961). Some factors concerned in differential nerve block by local anaesthetics. *J. Physiol.* 157 565–580. 10.1113/jphysiol.1961.sp006743 13727924 PMC1359995

[B35] Robertson A. Day B. Pollock M. Collier P. (1993). The neuropathy of elderly mice. *Acta Neuropathol.* 86 163–171. 10.1007/BF00334883 7692694

[B36] Sagie I. Kohane D. S. (2010). Prolonged sensory-selective nerve blockade. *Proc. Natl. Acad. Sci. U.S.A.* 107 3740–3745. 10.1073/pnas.0911542107 20133669 PMC2840479

[B37] Sheets M. F. Hanck D. A. (2007). Outward stabilization of the S4 segments in domains III and IV enhances lidocaine block of sodium channels. *J. Physiol.* 582(Pt 1), 317–334. 10.1113/jphysiol.2007.134262 17510181 PMC2075305

[B38] Silva C. B. Groppo F. C. Santos C. P. Serpe L. Franz-Montan M. Paula E. (2016). Anaesthetic efficacy of unilamellar and multilamellar liposomal formulations of articaine in inflamed and uninflamed tissue. *Br. J. Oral Maxillofac. Surg.* 54 295–300. 10.1016/j.bjoms.2016.01.005 26826985

[B39] Tabatabai M. Booth A. M. (1990). Effects of lidocaine on the excitability and membrane properties of the nerve cell soma. *Clin. Physiol. Biochem.* 8 289–296. 2132163

[B40] Tasaki I. (1953). *Nervous Transmission.* Springfield, IL: Charles C. Thomas.

[B41] Vyas K. S. Rajendran S. Morrison S. D. Shakir A. Mardini S. Lemaine V. (2016). Systematic review of liposomal bupivacaine (Exparel) for postoperative analgesia. *Plast. Reconstr. Surg.* 138 748e–756e. 10.1097/PRS.0000000000002547 27673545

[B42] Weiniger C. F. Golovanevski L. Domb A. J. Ickowicz D. (2012). Extended release formulations for local anaesthetic agents. *Anaesthesia* 67 906–916. 10.1111/j.1365-2044.2012.07168.x 22607613

[B43] Weiss G. (1901). Sur la possibilité de rendre comparables entre eux les appareils servant à l’excitation électrique. *Arch. Ital. Biol.* 35 413–446.

[B44] Wild B. M. Morris R. Moldovan M. Krarup C. Krishnan A. V. Arnold R. (2018). *In vivo* electrophysiological measurement of the rat ulnar nerve with axonal excitability testing. *J. Vis. Exp.* 6:132. 10.3791/56102 29443059 PMC5912375

[B45] Wong F. Fan L. Wells S. Hartley R. Mackenzie F. E. Oyebode O. (2009). Axonal and neuromuscular synaptic phenotypes in Wld(S), SOD1(G93A) and ostes mutant mice identified by fiber-optic confocal microendoscopy. *Mol. Cell. Neurosci.* 42 296–307. 10.1016/j.mcn.2009.08.002 19683573

[B46] Wu C. L. Raja S. N. (2011). Treatment of acute postoperative pain. *Lancet* 377 2215–2225. 10.1016/S0140-6736(11)60245-6 21704871

[B47] Yin Q. Ke B. Chen X. Guan Y. Feng P. Chen G. (2016). Effects of liposomes charge on extending sciatic nerve blockade of N-ethyl bromide of lidocaine in rats. *Sci. Rep.* 6:38582. 10.1038/srep38582 27924842 PMC5141481

[B48] Zaslansky R. Rothaug J. Chapman C. R. Backstrom R. Brill S. Fletcher D. (2015). PAIN OUT: the making of an international acute pain registry. *Eur. J. Pain* 19 490–502. 10.1002/ejp.571 25132607

